# ‘I Didn’t Have the Language Then’—A Qualitative Examination of Terminology in the Development of Non-Binary Identities

**DOI:** 10.3390/healthcare11070960

**Published:** 2023-03-28

**Authors:** Nat Thorne, Zoe Aldridge, Andrew Kam-Tuck Yip, Walter Pierre Bouman, Ellen Marshall, Jon Arcelus

**Affiliations:** 1 The Institute of Mental Health, The University of Nottingham, Nottingham NG7 2RD, UK; 2 The Nottingham Centre for Transgender Health, Nottingham NG1 3AL, UK; 3 School of Sociology and Social Policy, The University of Nottingham, Nottingham NG7 2RD, UK; 4 Bellvitge Biomedical Research Institute (IDIBELL), University of Barcelona, 08007 Barcelona, Spain

**Keywords:** non-binary, gender diverse, terminology, thematic analysis, transgender

## Abstract

Introduction: Identities that lie outside of exclusively male and female, such as non-binary and genderqueer, have become increasingly more prevalent and visible within recent years. However, to date, the role of terminology in the development of such gender identities has been under-researched. This study aims to: (1) Examine what role terminology plays in coming to identify as non-binary. (2) Explore the continuing importance of terminology once a non-binary identity is established. Methods: This study uses thematic analysis on data produced from interviews with 16 participants who self-selected for the study and were recruited from several transgender and LGBTQ+ organisations on the basis that they identified outside the gender binary of male and female. Results: The analysis uncovered several key themes and sub-themes relating to terminology choice, encountering new terms and the process of identifying with new terminology, as well as becoming visible and understood by others. Conclusions: This study found that terminology is not only central in coming to identify as something other than exclusively male and female, it also remains an important factor when it comes to making a non-binary identity visible to others.

## 1. Introduction

In recent years, identities that fall outside the gender binary of male and female have become more widely recognised. There are now a variety of terms used to describe these identities, such as genderqueer or non-binary, in addition to new terms constantly emerging [1,2,3]. This study uses the term non-binary as an umbrella term to describe all identities that fall outside of the gender binary.

Mainstream views of gender identity development focus heavily on the binary of male and female. Generally, it is accepted that children can ‘declare’ themselves either male or female by the age of around two or three [4,5]. The process of forming a gender identity is still not completely understood. Many leading theories suggest social learning and language are key factors [6], as well as viewing gender concepts in action through parents, adults and peers [7]. Although language plays a significant role in this development, acquiring and understanding gendered words is not sufficient; seeing gendered concepts in action is also vital. Within more specific development theories relating to transgender identities, language and terminology also play a role. Devor (2004) [8] describes how encountering the concept of being transgender leads a person to question their gender identity. Gagné et al. (1997) [9] describe how interaction with role models is an important part of identity discovery for trans male and trans female individuals, far more than just encountering the word transgender for the first time. These theoretical concepts are formed around gender binary-based ideas where a trans person moves from male to female, or vice versa, and do not include those who identify as non-binary. With the introduction of the internet, there is easier access to information which helps individuals explore their gender identity [10].

Language is one of the many areas which is dominated by binary gender, including different pronouns, given names and even the gender of inanimate objects such as ships [11]. A great deal of language is structured around the basics of a binary system rooted in gender. However, the emergence of new gender terminology, via such media as social media and community groups, has helped give a name to those who feel their gender does not align exclusively with either side of the gender binary. Encountering new terminology to describe non-binary identities has been suggested as the trigger that initiates a person’s realisation that their gender does not align with the one assigned to them at birth [12].

### Current Study

Little is known about the role terminology plays in gender identity formation for non-binary individuals. In addition, the continuing role of terminology after establishing a non-binary identity has also not been investigated. This study aims to: (1) Examine what role terminology plays in coming to identify as non-binary. (2) Explore the continuing importance of terminology once a non-binary identity is established.

## 2. Materials and Methods

The sample for this study consisted of participants who self-selected on the basis that they identified as any descriptor other than male or female, lived in the UK and were over 18. Whether participants had or had not undertaken gender-affirming medical treatments (GAMT), such as hormone therapy or surgery, or intended to in the future, did not affect their eligibility to participate in the study. This study aimed to recruit enough participants to reach saturation level. The definition of saturation used for this study is when new information fails to emerge from interviews [13]. A call for participants was placed on the Facebook group of a local transgender support organisation, based in Nottingham (Notts Trans Hub). In addition, several university LGBTQ+ networks shared the advert with their members. Snowball sampling was also used. The majority of participants were recruited from Twitter (8 participants) as well as from the post on a local charity’s Facebook page (4 participants). There were also 2 participants from snowball sampling and 2 from university LGBT+ networks.

The primary researcher is a non-binary identifying white British person. The primary researcher carried out the interviews, transcription, and analysis. The results were triangulated by a group of researchers consisting of two white European gay cisgender males, a British cisgender gay male, a white asexual transgender British female, and a white cisgender heterosexual British female.

Ethical approval was granted from the MASKED FOR REVIEW Psychiatry and Applied Psychology Department. To aid the recruitment process, a basic website was constructed which contained a copy of the participant information as well as a list of support organisations. The website also carried an electronic consent form that conformed to the BPS Code of Human Research Ethics as well as eiDAS Regulation No. 910/2014. A short demographic survey was emailed to the participants before the interview. Although none of the questions were mandatory, all participants answered the survey in full. Interviews took place between August 2019 and June 2020. The interviews were carried out either by telephone (*n* = 6), Skype (*n* = 7), or face to face (*n* = 3). The end of the study coincided with the COVID-19 nationwide lockdown, and so the final 3 interviewees were only given the option of either a telephone or Skype interview.

All interviews were semi-structured. The questions for the interview were generated by discussions with the research team, approved as part of the ethics procedure and informed by a systematic review which had previously been carried out and published by the lead researcher (MASKED FOR REVIEW). Transcription was completed in clean verbatim by the primary researcher, using Otranscribe software [14].

Thematic analysis as laid out by Braun and Clarke, 2006 [15] was used. The study took an inductive approach with the findings very much driven by the participants’ responses. An a priori codebook was not used, and all codes were drawn from the material. The analysis also took a latent approach, reading into the data to find relevant meanings rather than an explicit reading of the content. The data were coded using NVivo 12 software and triangulated by two other researchers who read through the themes, sub-themes, and example quotes before discussing the relevance of the themes and quotes concerning the research question. In addition, the themes and sub-themes were member-checked via a small email group of study participants who had opted into this stage of the process when asked at the end of the interview. The participants commented on whether the themes that were identified resonated with their own experiences [12]. The feedback agreed with the themes and so no further changes were needed.

## 3. Results

In total, 16 participants were interviewed. Researchers felt that saturation had been reached as no new data were emerging from the interviews and this size is in line with the recommendations for thematic analysis [16]. Specific data relating to each participant can be found in Table 1.

Responses from the demographic questionnaires showed that 11 of the sample were assigned female at birth (AFAB), while 5 were assigned male at birth (AMAB). The most popular identifier used by the sample was non-binary, with 11 participants stating that was their primary identifier. Three participants used more than one identifier and explained they would use different identifiers in different situations. Participants were aged between 21 and 45 (M = 30.31, SD = 8.22). The majority of the participants identified as white British, with one participant identifying as ‘Caucasian Jewish’ and one identifying as ‘White European’. In terms of education, 14 participants had gained a university degree or above, while two reported A Levels as their highest level of education. All participants stated that they identified with a non-heterosexual sexuality, with the majority identifying as pansexual.

The following three key themes were identified: (1) Encountering non-binary terminology fosters a feeling of validation. (2) Exploring the word ‘trans’ leads to a stronger association with the term. (3) Using terminology in order to make gender identity visible to others. Each of these key themes contained a series of sub-themes, which are detailed in Table 2.

### 3.1. Encountering Non-Binary Terminology Fosters a Feeling of Validation

Participants described how they were brought up in a society which only recognised two gender identities—male and female—and that they initially identified as their gender assigned at birth, despite feeling it was not quite right for them.

#### 3.1.1. Encountering Terminology through the Internet and LGBTQ+ Friends

Participants encountered terminology for non-binary identities in various ways. Many of the participants first experienced words for identities other than exclusively male or female on the internet. For example, Participant 8 said: “I probably heard the term used a couple of times and probably in various places like Tumblr, Facebook, social media basically”. Other participants could be more exact about how the internet first introduced them to the new terms. Participant 14 recalled: “So it was during the sixth form that I found that non-binary was a thing because there was that whole joke about Tumblr, with people identifying as whales or whatever”.

Access to the LGBTQ+ community had already been established, as every participant in the study had identified with a sexuality other than heterosexual before identifying as non-binary. It was in LGBTQ+ communities that some mentioned they had first encountered terms for non-binary identities, such as Participant 1 who said: “I heard about genderqueer somewhere in queer communities, which I already felt a part of due to my sexuality”. A number of the participants specified that their first encounter with words such as non-binary and genderqueer, as well as those who identified with these terms, was through their university’s LGBTQ+ society.

“I became one of the committee members for my student society—the bisexual rep—so I just took the time to educate myself as much as possible … there was also a really interesting community that was really quickly educating me about gender identity.”

(Participant 6)

#### 3.1.2. Not Instantly Identifying with Non-Binary Terms

On encountering the words, no participants instantly identified with them. Instead, it was a trigger which led to a much longer process of learning about and identifying with identities outside of male and female. For example, Participant 6 said: “It was a growing awareness. There wasn’t a thunderbolt when I thought ‘ah that’s me, that really describes my experience well’ … I think it evolved slowly”.

#### 3.1.3. Exploring the Meaning of Non-Binary Words via LGBTQ+ Friends

Many participants mentioned that it was within conversations that they gained a deeper understanding of what gender identity meant, and had the opportunity to ask questions and explore ideas.

“It wasn’t something that I really read about so much as like, a really long gradual realisation through things like talking to other genderqueer people about their experiences and what being genderqueer meant to them.”

(Participant 2)

After discovering the new terminology and then discussing and learning about alternative gender identities with other trans/non-binary individuals, participants began to accept that their own identity may be non-binary. These discussions prompted the participants to begin using gender-neutral pronouns and identity labels other than male and female when describing their gender to others.

#### 3.1.4. New Terminology Feels Validating

The decision to move away from using terminology related to their sex assigned at birth was described as a validating experience by all participants. Participant 11 explained: “… Embracing those labels has been enormously important in terms of me being able to actually find joy in gender and gender expression”. Participant 7 also found discovering words to describe their identity validating: “It was actually a really nice moment because I’d finally found an explanation, or at least a description. I wasn’t just weird and there were other people like me”.

### 3.2. Exploring the Word Trans Leads to a Stronger Association with the Term

The interviews also explored participants’ association with the word ‘trans’. Mirroring the experience of adopting non-binary identities, none of the participants felt they could place themselves under the trans umbrella when they first heard the word. Participants generally had a basic understanding of the word trans from an early age but described how, to them, it meant changing from one binary gender to another and always involved medical treatment. Finding out the term had a wider meaning meant that some came to see themselves as trans.

#### 3.2.1. Not Earned the Term ‘Trans’

Participant 11 said: “I think partly I don’t feel entitled to claim it [trans] as somebody who is always read as a cis woman anyway and who doesn’t experience certain types of abuse”. Other participants who did not identify as trans stated they were open to the idea of exploring trans as a part of their identity. For example, Participant 3 said: “I think of myself as an ally. I am interested in what opens up when I use the word trans for myself, but I feel like, at this point in time, that refers to a struggle that I can’t claim”.

#### 3.2.2. Re-Defining the Meaning of the Word ‘Trans’

Some participants said they adopted the label trans after talking to other trans people and hearing them accept non-binary people as trans, while for others there was a process of recognising that the word had a wider definition than they first thought and that, in fact, their own identity aligned with that definition:

“There was definitely a phase where I felt I wasn’t trans enough, and especially at the point where I wasn’t sure I wanted to have any surgery or hormone therapy or change my name or change my pronouns. Now I am pretty much of the opinion that none of that is important because what makes you trans is the fact that you identify with a different gender to the one you were assigned at birth.”

(Participant 12)

Several participants said that conversations with other trans people opened up a new meaning of the word for them. For example:

“And after hearing those words from other trans people again and again, ‘you are trans if you say you are …. you don’t need to prove anything to anyone’; I said ‘well, to hell with it then … I am trans’.”

(Participant 15)

#### 3.2.3. Identifying Beneath the Trans Umbrella Feels Useful

For those who did eventually see themselves under the trans umbrella, participants said the term was useful when discussing their own identity to others. Aligning themselves with the trans community also felt very validating to them. For example:

“I think trans is a really useful word because it’s kind of used as an umbrella term or a catch-all …. I may look very obviously female or very obviously male or perhaps a way people aren’t going to know what my gender necessarily is. Trans is just non-committal and leaves me some wiggle room.”

(Participant 6)

### 3.3. Using Terminology to Make Gender Identity Visible to Others

Participants described how being acknowledged as non-binary is difficult in a society that considers gender to be binary. This meant that participants had to make compromises in the terminology they used in order to be legible to the rest of society.

#### 3.3.1. Use Non-Binary as It Is the Best-Known Term

A few participants explained how they felt that non-binary is the most widely-known term outside of the LGBTQ+ community, and that this is why they chose to use it. Participant 7 said: “So I use non-binary just because that’s the term that seems to be most popular at the moment”.

Although some participants felt that non-binary was a suitable term for them, several participants described how they had accepted the term non-binary, and started to use it as one of their identifiers, out of necessity in order to have their identity recognised. “Non-binary is the quickest way to explain the pronouns, the fact that I’m not using my given name …. The word itself I find really clumsy.” (Participant 7).

#### 3.3.2. Use More Than One Term

Several of the participants said that they tend to use more than one term to describe their gender identity, and their choice depends on the social context they are in. In addition, many of the participants said they had identified with other terms before they settled on the ones they currently use. Participant 16 said that they have gone through several labels to describe their identity: “There was a time when I used genderqueer. I also remember androgynous probably being the main starting point. Androgynous then evolved to non-binary having realised that I wasn’t as genderless as I had initially thought.” Participant 12 described how they were concerned that the language used within the trans community would not be recognisable to those outside of it.

“It concerns me that people aren’t going to understand outside the community what I mean by non-binary, so even just adding the word trans, people are going to have some clue that I am somewhere under the trans umbrella and that’s helpful to me.”

(Participant 12)

## 4. Discussion

This study aimed to: (1) Examine what role terminology plays in coming to identify as non-binary. (2) Explore the continuing importance of terminology once a non-binary identity is established.

Participants talked extensively about discovering terminology related to non-binary identities, and these terms reflected their own experiences of gender. Sex assigned at birth currently comes with an inherited terminology such as boy/girl, male/female that renders any identity other than male and female as linguistically invisible. Many developmental theories of gender place language as central to a person’s understanding of their social grouping [5,6,17]. As philosopher Ludwig Wittgenstein described it: “the limits of my language mean the limits of my world” [18]. The relative invisibility of terms outside of the gender binary meant that participants did not question whether their identity fell outside the gender binary of male and female.

For some participants, finding the right terminology involved searching online for terms describing identities beyond the gender binary. However, for most participants the internet was not the only resource where new information on non-binary terminology was sought. Participants also gathered vital information and terminology via the LGBTQ+ community, of which all participants expressed they already felt a part due to their non-heterosexual sexual orientation. Conversations with other members of the LGBTQ+ community gave participants a space to ask specific questions and to understand gender through the experiences of others. This supports the work of Gagne et al., 2017 [9], which placed interaction with peers as an important part of coming to identify as transgender.

The results here expand on the prior work of Devor, 2005 [8], who suggested that encountering the concept of transgender identities, and learning there is a word for this feeling, is the first developmental step for a transgender person. In a similar way to Devor’s description of accepting a binary transgender identity, participants in this study did not immediately identify with non-binary terms, but spent a period of time learning about the terminology in practice and what the words meant before coming to identify with them.

Participants also discussed at length their association with the term trans. For many participants, even after they had started identifying outside of exclusive male or female, accepting that their identity could be described as trans took time. Most described a feeling of ‘not deserving’ the label trans. Darwin (2020) [19] discussed non-binary individuals feeling they have not earned the label of trans as it still mostly viewed as a binary and medical-based concept. This leads to the assumption that medicalisation and a struggle against being ‘born in the wrong body’ are very much essential parts of being trans, and this was reflected in the discussions with the participants in this study. However, encounters and discussions with trans and gender non-conforming individuals seemed to widen some of the participants’ views of what it meant to be trans, and allowed them to feel as though their own identity fitted underneath the umbrella. It should be noted that some participants in the study did not see themselves as trans. This could either be because they were earlier in the process of identity discovery or it could mean, for some, the word trans still does not represent their identity. Alternatively, with identities outside of exclusively male and female representing such a heterogeneous group, there may be other reasons for them not identifying as trans, such as feeling their identity is still closer to their sex assigned at birth, or that they present in a very cis way.

Terminology was described as an important tool participants used to make their identity visible and understood to others. However, making their identity known to others in a society which generally only recognises binary gender identities meant that participants often felt the need to compromise, describing themselves in terms which were not ideal. Many participants went on to explain how non-binary did not fully represent their gender identity. However, the prevalence of non-binary as an identifier made participants feel as though they needed to adopt this term to be acknowledged. The term non-binary also references the existence of a binary, which participants suggested may make it more palatable to society. Gendered terminology is currently dismissive of any identities outside of male and female; therefore it seems to make sense that participants would find a way to modify and use the existing terminology to describe themselves; by keeping the binary intact, but describing themselves as something ‘other’ to it.

This study establishes that terminology is central, not only in coming to identify outside of the binary, but also after a non-binary identity has been established. Terminology is also essential in the healthcare of trans and gender diverse individuals [20]. The emergence of non-binary identities has been viewed by some as a call to reject gender labels altogether, or degendering [21]. Rather than calling for fewer labels, the participants here desired a much wider range of terminology. In addition, participants felt as though their identity needed to fit within the current definition of certain popular terms, rather than words being chosen because they fitted that participant’s inner feeling of identity. Currently, the role of terminology in gender is to create a definition with expectations of behaviour, appearance and other aspects of identity which divide society into pre-defined categories. The participants in this study seemed to be calling for an alternative system, where the correct terminology is chosen in response to a person’s gender identity and inner feeling of gender. The social response to this is not easy. Currently, systems in society are streamlined to include just two groups. Everything from toilets to sports are easily navigated if society only has two pre-defined groups. In order to change and accommodate alternative identities, much work will have to be carried out to undo gender.

Participants also felt that words that define people outside the binary were essential in the process of identity development. The terminology provided a sense of validation of their gender identity and a feeling of belonging. In spite of this, the terms male and female were not sufficient to accommodate the participant’s identity, no matter how much the categories expand in order to include non-normative genders [21]. This would create a very heterogeneous group in which the nuance of the participant’s identity would get lost. This mirrors the participants’ current issue with the term non-binary, which also creates a group which is too wide to define the very nuanced feelings of gender that participants experienced. While moving towards a new identity was important, equally important was leaving behind a previous identity.

Legal recognition of identities beyond male and female is an important step in making such identities visible. Legislation would also offer individuals a legitimate claim to have their identity recognised by others. Naming this new group will, however, be a task needing much thought, research and consultation, as current popular terms such as non-binary are not ideal for all people who identify outside of male and female.

A limitation of this study is that it fails to capture the experiences of a more diverse range of participants. The cohort was exclusively white and had a high education level; therefore, the findings cannot be generalised to the non-binary population. This could possibly be due to the recruitment methods, and the networks within which the study was advertised. It could also suggest that those who have been students have been exposed to more information regarding gender, through such groups as LGBTQ+ university societies. Therefore, the high level of education of the participants may affect the findings of the study, and caution should be exercised when interpreting the results. The link between education level and non-binary identities would make an interesting subject for further study. Future research could also aim to investigate those from more diverse ethnic and educational backgrounds, by targeting online support groups and forums specific to certain groups, or ones that have a larger membership. This study was also limited to those who live in the UK and speak English. Other languages bring differing linguistic restrictions and possibilities; examining the relationship between terminology and identities outside of male and female may be interesting in countries whose language depends more upon a binary and, conversely, in those whose language does not. In addition, a study looking at how participants use language to negotiate their identity within society would add a deeper level of understanding beyond terminology.

The study uncovered several key themes and sub-themes relating to terminology choice, encountering new terms and the process of identifying with new terminology as well as becoming visible and understood by others. This study establishes that terminology is central to identifying as something other than exclusively within the gender binary, and provides validation of gender identity and a sense of belonging. In addition, language remains important after a non-binary identity has been established, to make a non-binary identity visible. The lack of widely used terminology to describe non-binary identities, as well as society’s dependence on a binary gender system, makes identity a complex task to navigate for those who do not feel that their identity fits neatly into either male or female.

## Figures and Tables

**Table 1:** Participant Demographics.

Participant No	Age	ASAB ^a^	Ethnicity	Education Level	Gender Identity	Sexual Identity
1.	45	F	White British	Postgraduate	Genderqueer	Queer
2.	30	F	White British	Graduate	It’s complicated	Bisexual
3.	21	F	White British	Graduate	Non-binary	Queer
4.	39	F	Caucasian Jewish	Doctorate	Non-binary Transmasculine Gendervague	Queer
5.	26	M	White European	Doctorate	Non-binary	Bisexual Pansexual
6.	25	M	White British	Degree	Non-binary Trans	Bisexual
7.	44	M	White European	Doctorate	Non-binary	Pansexual
8.	22	F	White British	Postgraduate	Agender	Asexual
9.	25	M	White British	Graduate	Non-binary	Pansexual
10.	41	F	American	Doctorate	Gender non-aligned	Queer
11.	33	F	White British	Postgraduate	Non-binary	Queer
12.	30	F	White British	Graduate	Non-binary Trans	Bisexual
13.	21	F	White British	A Levels ^b^	Non-binary	Asexual
14.	21	F	White British	A Levels	Non-binary	Pansexual
15.	33	F	White British	Postgraduate	Agender	Pansexual
16.	29	M	White British	Postgraduate	Non-binary	Pansexual

a = Assigned sex at birth. b = A Levels are the highest school qualifications in the UK.

**Table 2:** Themes and subthemes.

Main Theme	Subthemes
1. Encountering gender diverse terminology fosters a feeling of validation	1.A Encountering new terminology though the internet and LGBTQ+ friends 1.B Not instantly identifying with gender diverse terms 1.C Exploring the meaning of gender diverse words via LGBTQ+ friends and therapy 1.D New terminology feels validating
2. Exploring the word trans leads to a stronger association with the term	2.A Not earned the word trans 2.B Re-defining the meaning of the word trans 2.C Identifying beneath the trans umbrella feels useful
3. Using terminology to make gender identity visible to others	3.A Use non-binary as it is the best-known term 3.D Using more than one term

## Data Availability

Due to the nature of this research, participants of this study did not agree for their data to be shared publicly, so supporting data are not available.
